# Reliable Adaptive Data Aggregation Route Strategy for a Trade-off between Energy and Lifetime in WSNs

**DOI:** 10.3390/s140916972

**Published:** 2014-09-11

**Authors:** Wenzhong Guo, Wei Hong, Bin Zhang, Yuzhong Chen, Naixue Xiong

**Affiliations:** 1 College of Mathematics and Computer Science, Fuzhou University, Fuzhou 350116, China; E-Mails: fzugwz@163.com (W.G.); fzuerhw@gmail.com (W.H.); zhangb2366@163.com (B.Z.); 2 Fujian Provincial Key Laboratory of Networking Computing and Intelligent Information Processing, Fuzhou University, Fuzhou 350116, China; 3 School of Computer Science, Colorado Technical University, Colorado Springs, CO 80907, USA; E-Mail: nxiong@coloradotech.edu

**Keywords:** wireless sensor networks, mobile security, route, data gathering, data aggregation, particle swarm optimization

## Abstract

Mobile security is one of the most fundamental problems in Wireless Sensor Networks (WSNs). The data transmission path will be compromised for some disabled nodes. To construct a secure and reliable network, designing an adaptive route strategy which optimizes energy consumption and network lifetime of the aggregation cost is of great importance. In this paper, we address the reliable data aggregation route problem for WSNs. Firstly, to ensure nodes work properly, we propose a data aggregation route algorithm which improves the energy efficiency in the WSN. The construction process achieved through discrete particle swarm optimization (DPSO) saves node energy costs. Then, to balance the network load and establish a reliable network, an adaptive route algorithm with the minimal energy and the maximum lifetime is proposed. Since it is a non-linear constrained multi-objective optimization problem, in this paper we propose a DPSO with the multi-objective fitness function combined with the phenotype sharing function and penalty function to find available routes. Experimental results show that compared with other tree routing algorithms our algorithm can effectively reduce energy consumption and trade off energy consumption and network lifetime.

## 1. Introduction

Wireless Sensor Networks (WSNs) are one of the most important technologies changing the world in that such networks can be used in variety of applications, such as environment monitoring, military surveillance and object tracking, disaster area relief, industrial control and seismic monitoring. The basic function of a WSN is collecting and returning data from each sensor node in each respective monitored area where data may be highly correlated. Data gathering is a key operation for WSNs to extract useful information from the operating environment. Recent studies [1–5] show that data aggregation, a process dealing with several data to obtain what is more suitable for user needs, is tremendously useful in eliminating data redundancy and reducing the communication load. WSNs are generally energy constrained, because wireless sensor nodes are powered by batteries and usually deployed in some harsh environments which make it unrealistic to replace the batteries. Due to inherent resource and computing constraints, mobile security presents the most challenging task in designing secure routing protocols for WSNs.

There some security requirements which are typically studied in WSNs: confidentiality, integrity, authentication and availability [6]. The order in which these security requirements are studied is important, because it reflects the significance that researchers place on them in achieving and supporting security. Studying the security requirements based on their importance will allow researchers to gain a better understanding of the security aspects that they should focus on.

The availability and reliability are the most important security requirements in critical WSN applications. Availability refers to the fact that the services and information can be accessed at the time that they are required. This means that the network must provide reliable service to ensure that the data will be transmitted to the destination accurately. Availability is the basic requirements relative to other security attributes. That is to say the availability and reliability are placed at a higher rank in the security requirements chain for the reason that if sensor nodes get disabled or cannot exchange packets, any other security requirements that have been established will make no sense [7].

The use of dynamic routing can diminish the effect of security attacks that target the availability and resilience of the network. Regular data aggregation in WSNs is the operation whereby sensor nodes collect and transfer the data to the sink node. The aggregation typically follows a tree topology rooted at the sink. Each leaf node (sensor node) will deliver the data to its parent node until it reaches the sink. Dynamic Routing with data aggregation aims to find the optimum network topology for minimum energy consumption and maximum network lifetime in order to bypass some failed nodes which may have been attacked by an adversary and establish a reliable route to deliver the data [8]. Combined with the in-network processing, WSN keeps the cability of flexibility when it suffers attacks from adversaries. All of the above support and maintain the availability of the network and the provided services.

In our previous work [9], we have included aggregation cost in [10,11] as another dimension to the space of routing optimization for correlated data, which is a multi-objective problem. Combined with the Pareto method, a heuristic algorithm based on discrete particle swarm optimization (DPSO) is designed to find approximate solutions for it. Compared with [9], the major contributions of this study can be summarized as follows:

(1) Like [9], we also adopt the same strategy of discrete particle swarm optimization with crossover and mutation operators. We adopt the Purfer encoding scheme and design uniform crossover and random two-point exchange mutation operators to avoid generating infeasible solutions.
(2) We apply a phenotype sharing function of the objective space in our algorithm for the establishment of reliable routes (similar to the approach in [9]). Since it is a multi-objective optimization, a fitness function based on the phenotype sharing is designed considering both the Pareto dominance and the neighborhood density of the objective space. The algorithm based on the phenotype sharing function obtains a better approximation of a true Pareto front.
(3) Different from [9], in order to declare the validity of encoding scheme, we prove that the Prufer sequence can satisfy well the principles of non-redundancy, completeness and soundness. Therefore it can not only reduce the redundancy of the search space, but also improve the search efficiency, and thereby enhance the performance of the algorithm.
(4) For achieving more Pareto feasible solutions and improving the convergence speed, we further introduce the penalty function to convert the constrained optimization problem into a non-constrained one. The fitness function based on penalty mechanism is proposed. We also extend the analysis about the effect of penalty function on the optimal problem. We have conduct comprehensive comparisons with four different penalty functions to study the performance of penalty function in DPSO.

The rest of this paper is organized as follows: in Section 2, the related work is presented. In Section 3, the system model and problem formulation are described in detail, and then we present our proposed methodology and strategy in Section 4. In Section 5, we compare our algorithm with other algorithms and evaluate their performance. Finally, concluding remarks are made in Section 6.

## 2. Related Work

Energy consumption is an essential factor as the nodes which are battery operated devices and not rechargeable energy ones fail easily due to the energy depletion. Therefore, energy consumption becomes a primary concern in most WSN applications. A WSN protocol should typically ensure that connectivity in a network is maintained, even in the presence of node failure (e.g., due to some security attack or other reason like energy depletion). In the existing literature, a number of issues relating to routing design and security issues have been reviewed.

Energy-efficient routing algorithms for data gathering are a major concern in WSNs. Routing tree structures are adopted in many previous works [10,12–15] to collect data: a sensor node transmits its data and the data from its child nodes to its parent node. In [12], the authors considered the problem of correlated data gathering by a network with a sink node and a tree-based communication structure, and proved that minimum-energy data gathering problem is NP-complete and declared that the optimal result is between Shortest Path Tree (SPT) and Traveling Salesman Problem (TSP). In [13], the authors proposed an optimal algorithm called MEGA for foreign-coding and an approximate algorithm called LEGA for self-coding. In MEGA, all nodes firstly send their gathered data to the sink node via the Minimum Spanning Tree (MST) and then each encoding node sends its respective encoded data to the sink node through the SPT rooted at the sink. In LEGA, the sink node broadcasts its packet to its neighbor nodes and each node sends its data to the sink node by the constructed Shallow Light Tree (SLT). By constructing the SLT, LEGA achieves a $2(1+\sqrt{2})$-approximation of the optimal data gathering route. Khan *et al.* [14] proposed a scheme, called NNT, which was a variant of using greedy algorithm to construct a minimum Steiner tree. NNT builds a slightly suboptimal tree with low energy complexity, and it is proved that NNT can be used to design a simple dynamic algorithm for maintaining a low-cost spanning tree.

However, these above literatures only pay attention to transmission cost in building the routing tree, and neglect the cost of aggregating correlated data. In some practical applications, such as image aggregation, the aggregation cost may be greater than the transmission cost [16]. Therefore, in addition to transmission cost, aggregation costs can significantly affect routing decisions when data aggregation is involved. Luo *et al.* [10] put forward the MFST algorithm, which was applied to collect data with aggregation by an energy-efficient method in WSNs. MFST takes both transmission cost and aggregation cost into account, and chooses aggregation nodes based on the quantity of data generated by each node. Luo *et al.* [15] further proposed an improved MFST algorithm called AFST. AFST dynamically decides whether to proceed with data aggregation when each relay node transmits data, rather than merely optimizing the data transmission route.

## 3. System Model and Problem Formulation

### 3.1. Network Model

**Figure 1.**
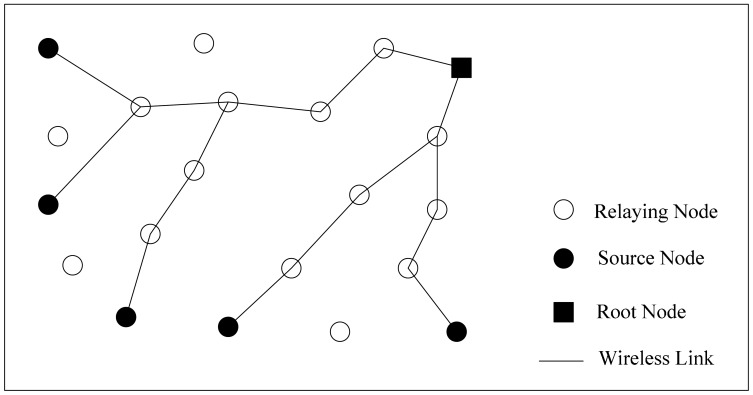
Network model diagrams.

In this paper, we consider a wireless sensor network composed of *n* nodes which are distributed uniformly and randomly in the areas of monitored regions as shown in Figure 1. Since the typical mode of communication in data aggregation involves multiple data source nodes and one sink node [17], without lost of generality we assume there are *k* (*k* ≤≤ *n*) source nodes and one sink node. Node *u* can receive the data from node *v* if node *u* is within the communication range of node *v*; otherwise, they have to communicate with each other through multi-hop wireless links [18,19]. We model a WSN as an undirected graph *G*(*V,E*), where *V* is a finite set of sensor nodes, and *E* is defined as the wireless connection between nodes [20–24].

### 3.2. Correlation and Data Aggregation

As mentioned previously, data from multiple child nodes along the routing tree can be aggregated in order to reduce the communication load of network. The aggregation process is an essential data compression process and the compression ratio is related to data correlation and redundancy. Due to the uncertainty of the ratio in different application scenarios [7], we use an abstract parameter ρ to denote the data reduction ratio due to aggregation. To be more specific, if node *u* is a child node of node *v* in the constructed routing tree and *u* transmits its data to *v*, we can summarize the aggregation function at node *v* as:

(1) $$\omega (v)=\left(\omega \left(u\right)+\varpi \left(v\right)\right)\left(1-\rho_{uv}x_{uv}\right)$$

where *ϖ*(*ν*)and *ω*(*ν*) denote the data amount of node *v* before and after aggregation respectively, and *x_uν_* ∈ {0,1} denotes whether aggregation process occurs between node *u* and node *v*. That is to say, if node *v* is an aggregation point, the data amount of *v* after aggregating income data of node *u* is *ω*(*ν*) = (*ω*(*u*)+*ϖ*(*ν*))(1−*ρ_uv_*); otherwise, *ω*(*ν*) = (*ω*(*u*)+*ϖ*(*ν*)).

### 3.3. Energy Model

Here, we will jointly consider two aspects of costs: communication cost and aggregation cost. We will use the following radio communication model [25,26] to calculate the energy consumption for sending and receiving data. The energy model can be respectively represented by the Formula (2) and Formula (3):

(2) $$E_{T}\left(u,v\right)=\left(\alpha +\beta *{d_{uv}}^{2}\right)\times \omega \left(u\right)$$

(3) $$E_{R}\left(u,v\right)=\alpha \times \omega \left(u\right)$$

where α is the energy consumed by each sending node to send each bit of data, or each receiving node to receive each bit of data. β is the energy consumption in the amplification circuit for forwarding each bit of data. *d_uv_* is the distance between node *u* and node *v. ω*(*u*) is the data amount transmitted from node *u*, so for edge *e* = (*u, v*), the communication cost *t* (*e*) of edge *e* is given by:

(4) $$t\left(e_{uv}\right)=\left(2\alpha +\beta \times {d_{uv}}^{2}\right)\times \omega \left(u\right)$$

Here we discuss two node types: aggregation points and non-aggregation points. If node *v* is a non-aggregation point, it will merely consume energy to transmit data and receive data; if not, it should further consume energy to aggregate its own data and the data from its child nodes. That is to say, besides communication cost, in this paper we also include an aggregation cost model which is presented in [8]. We use Equation (5) to represent the cost for fusing the data of node *u* and *v*:

(5) $$f\left(e_{uv}\right)=q\times \left(\omega \left(u\right)+\varpi \left(v\right)\right)$$

where *q* indicates average unit aggregation cost and it is dependent on the type of data to be aggregated and data correlation. *ω*(*u*) and *ϖ*(*ν*) are data amount from *u* and *v* own data amount before aggregation.

### 3.4. Lifetime Model

Network lifetime [27] is concerned with the period in which the network can maintain its desired functionality. It can be defined as the time till the first node in the network dies, called nodal lifetime; or it can also be defined as the time till a certain proportion of the nodes die. We will use the former definition as sensor network lifetime in the subsequent discussions in the rest of this paper.

For each sensor node in the network, its energy consumption may involve many factors. For simplicity, here we neglect the impact of other secondary factors and only attach importance to three main factors: transmitting data, receiving data and aggregating data. Therefore for an undirected graph *G(V,E)*, the nodal lifetime of node *v*(*v*∈*V*) can be described as follows:

(6) $$l\left(G,v\right)=\frac{E_{r}\left(v\right)}{\sum f+\sum E_{T}+\sum E_{R}}$$

where *E_r_*(*v*) indicates residual energy of node *v*. ∑*E_T_*, ∑*E_R_* and ∑*_f_* are the energy of node *v* used to transmit data, receive data and aggregate data if it is a aggregation point. According to previous description, a network ends up with the first node depleting its energy, so we can easily formulate network lifetime as follows [28,29]:

(7) $$l\left(G\right)=\underset{v\in V}{\min}l\left(G,v\right)$$

From the above formulas, it can be obviously seen what kinds of factors are significant to the uneven energy consumption issue, which can help us to explore a more effective algorithm to extend network lifetime in the right direction.

### 3.5. Problem Formulation

Given an undirected graph *G*(*V,E*), source node set *S* and sink node *t*, we assume *G*′ is a connected subgraph of *G* and energy consumption of *G*′ is given by:

(8) $$E\left(G'\right)=\sum_{e\in E{'}_{f}}\left(f\left(e\right)+t\left(e\right)\right)+\sum_{e\in E{'}_{n}}t\left(e\right)$$

*E*′*_f_* is an edge set where the end node of each edge is an aggregation point. *E*′*_n_* is an edge set where the end node of each edge is a non-aggregation point.

Our first objective is to find a near-optimal subgraph G* that at least contains node set *S* and sink node *t* such that:

(9) $$G^{*}=\arg\min_{G'}E\left(G'\right)$$

It has been shown that the aforementioned problem is NP-hard [8]. Without considering the imbalance of energy consumption, the constructed routing tree may result in premature death of some nodes, energy hole problems [30] and so on. On the basis of (9), we further consider the remaining nodal energy and dynamically adjust the routing strategy to balance nodal energy, effectively prolonging network lifetime, so our second goal is to find a feasible subgragh *_G_*_′_ considering nodal remaining energy such that:

(10) $$\begin{array}{c} \min E\left(G'\right)\\ \max l\left(G'\right)\\ s.t.E\left(G'\right)\leq \varepsilon E\left(G^{*}\right) \end{array}$$

Notice that *ε*(epsilon) denotes maximal permissible times of energy consumption of *G*′ to G* and it is a variant that is set by us. Similar to the problem of finding an optimal subgraph G*, the above problem is also a NP-hard multi-objective optimization problem.

## 4. Algorithm

### 4.1. Basic Particle Swarm Optimization

Particle Swarm Optimization (PSO) is a population-based search problem where each particle is defined as a potential solution to a problem in a *D*-dimensional space. With the *i*th particle represented as *X_i_* = *(X_i1_, X_i2_,…,X_iD_)*, each particle adjusts its position to close to the minimum according to its own experience and that of neighboring particles. Each particle also maintains a memory (*pbest*) of its previous best position represented as *p_i_* = *(p_i1_, p_i2_,*…,* p_iD_)* and a velocity along each dimension represented as *V_i_* = *(V_i1_, V_i2_,*…*,V_iD_)*. In each generation, the *pbest* vector of the particle with the best fitness in the local neighborhood is designated as *p_gd_*. In each generation of early PSO versions, the particles are manipulated according to the following equation:

(11) $$v_{id}^{t+1}=w\times v_{id}^{t}+c_{1}r_{1}\left(p_{id}-x_{id}^{t}\right)+c_{2}r_{2}\left(p_{gd}-x_{id}^{t}\right)$$

(12) $$x_{id}^{t+1}=x_{id}^{t}+v_{id}^{t+1}$$

where *t* is the iteration index, *d* is the number of dimensions, *w* is inertia weight, *c_1_* and *c_2_* are acceleration factors, *r_1_* and *r_2_* are random numbers in the range [0…1].

### 4.2. Discrete Particle Swarm Optimization

Since the previously mentioned optimization objectives are NP-hard discrete problems, it is obvious that the standard PSO is not appropriate for those above problems for its continuous nature, so some modifications must be done to improve the standard PSO. Since the PSO algorithm was proposed by Kennedy *et al.* in 1995, several discrete PSO algorithms have been proposed, including the discrete PSO algorithm [31], the discrete PSO algorithm for the traveling salesman problem [32], the discrete PSO algorithm for the permutation flow shop sequencing problem with makespan criteria [33]. Inspired by our previous work [6], a discrete PSO is designed here to achieve the two optimization objectives mentioned above. Moreover, the problem (10) is also a complex nonlinear constrained optimization problem, so in this paper the penalty function combined with the phenotype sharing function is introduced to convert it to a non-constrained optimization problem and applied in the definition of fitness function.

#### 4.2.1. Representation of Particles

Due to robustness of the PSO algorithm, it does not demand rigorous representation of particles. A good representation can not only reduce the redundancy of the search space, but also improve the search efficiency, and thereby enhance the performance of the algorithm. Generally speaking, the selection of particle representation should comprehensively consider the following three main principles: non-redundancy, completeness and soundness.

*Definition 1 (Non-redundancy):* A one-to-one relationship between the particles in the encoding space and the potential solution in the problem space.

*Definition 2 (Completeness)*: Each point in the problem space (feasible solution) can become the phenotype of points in the particles' encoding space.

*Definition 3 (Soundness)*: Each particle in the encoding space must correspond to a potential solution in the problem space.

It is well known that it is difficult for an encoding scheme to meet all three principles above. In this paper, as our target is to construct an efficient routing tree structure, we adopt the Prufer sequence in [34] to represent a labeled tree *T* whose vertexes are numbered from 1 to *n*. We can easily construct the Prufer sequence according to the following procedure:

*Procedure: Encoding*
  Step 1: Let *j* be the smallest labeled leaf vertex in the *T*.
  Step 2: Set *k* to be the first digit in the Prufer sequence if *k* is incident to *j*.
  Step 3: Remove *j* and the edge which connects *j* and *k* from *T*.
  Step 4: Repeat above steps until only one edge is left and produce the Prufer sequence in order.
*Procedure: Decoding*
  Step 1: Let *P* be a Prufer sequence and *Q* be the set of all vertexes not included in *P*.
  Step 2: Let *j* be the vertex with smallest label in *Q* and *k* be the leftmost digit in *P*. Add the edge connecting *j* and *k* into the tree. Remove *j* from *Q* and *k* from *P*. If *k* does not occur anywhere in *Q*, put it into *Q*.
  Step 3: Repeat above steps till no digit is left in *P*.
  Step 4: If no digits remain in *P*, there are exactly two vertexes in *Q*. Add the edge connecting remaining vertexes into the tree.

**Figure 2.**
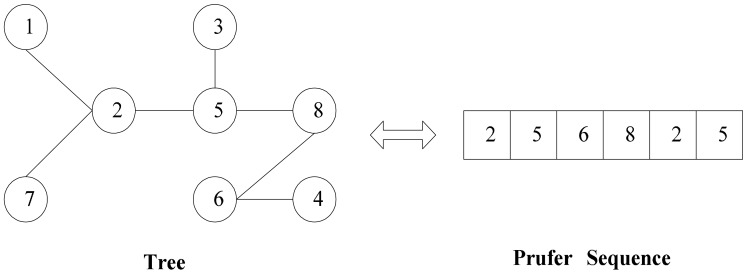
A tree and its Prufer sequence.

An example is given to illustrate above encoding and decoding procedures. The Prufer sequence corresponds to a tree on an 8-vertex complete graph which can be seen in Figure 2.

*Properties*: The Prufer sequence that represents a spanning tree of a complete graph can satisfy the principles of non-redundancy and completeness, but does not meet the principle of soundness.

*Proof*: Assuming that *T* represents a spanning tree of an *n*-vertex complete graph and *A* represents a non-leaf node of *T*. Since *A* is at least connected with two different nodes, the label of *A* must have appeared in the corresponding Prufer sequence when only one edge remains in the *T*. In reverse, the numbers, which appear in the Prufer sequence finally, are obviously not the leaf nodes of *T*, while the other numbers are the leaf nodes of *T*. Assuming that *B* represents the smallest number which has not appeared in the Prufer sequence, thus *B* is the leaf node which is incident to leftmost labeled vertex in the Prufer sequence. Then, we recursively consider following *n*-3 numbers and connect the remaining two vertexes finally. As the number of nodes not processed the rest of the code length by 2, we can always find out a smallest number which has not appeared in the remaining number of coding. Thus, any Prufer sequence can uniquely correspond to an unrooted tree. So the Prufer sequence can satisfy the principle of non-redundancy.

One of the classical theorems in graphical enumeration is Cayley's theorem which states that there are *n*(*n*−2) distinct labeled trees for a complete graph with *n* vertices. The Prufer sequence can use only a permutation of *n*−2 digits in order to uniquely represent a tree with n vertices where each digit is an integer between 1 and *n* inclusively. Moreover, we have already proved that any Prufer sequence can uniquely correspond to an unrooted tree, thus Prufer sequence with *n*−2 digits can totally represents *n*^(n−2)^ distinct labeled trees. So the Prufer sequence can satisfy the principle of completeness.

However, the problem (10) is a nonlinear constrained optimization problem, and there are some particles which may correspond to infeasible solutions, such as the actual energy consumption of some route trees after decoding may exceed the energy consumption constraint. So here the Prufer sequence cannot satisfy the principle of soundness.

To meet the principle of soundness, the penalty function combined with the phenotype sharing function is introduced to convert the nonlinear constrained optimization problem to a non-constrained one and applied in the definition of fitness function to ensure that all particles are feasible. And it will be elaborated in detail in subsequent sections.

#### 4.2.2. Discrete Procedure of PSO

The notion of mutation operator in GA [35,36] is incorporated into the first part of Equation (11)

(13) $$A_{i}^{t}=F_{1}(X_{i}^{t-1},w)=\left\{ \begin{array}{l} M(X_{i}^{t-1}),r_{1}<w\\ X_{i}^{t-1},\text{otherwise} \end{array}\right.$$

where *F_1_* indicates the mutation operator with the probability of *w*. The second and third parts of Equation (11) all adopted the notion of crossover operator in GA:

(14) $$B_{i}^{t}=F_{2}(A_{i}^{t},c_{1})=\left\{ \begin{array}{l} C_{p}(A_{i}^{t}),r_{2}<c_{1}\\ A_{i}^{t},\text{otherwise} \end{array}\right.$$

(15) $$X_{i}^{t}=F_{3}(B_{i}^{t},c_{2})=\left\{ \begin{array}{l} C_{g}(B_{i}^{t}),r_{3}<c_{2}\\ B_{i}^{t},\text{otherwise} \end{array}\right.$$

where *F_2_* and *F_3_* indicate the crossover operators with the probability of *c*_1_ and *c*_2_ respectively. Then the position of the *i*-th particle at iteration *t* can be updated as follows:

(16) $$X_{i}^{t}=F_{3}(F_{2}(F_{1}(X_{i}^{t-1},w),c_{1}),c_{2})$$

**Figure 3.**
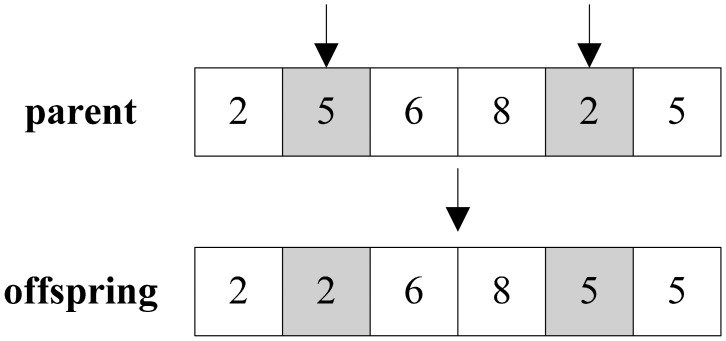
Illustration of mutation operation.

Though there are different crossover and mutation operators for different encoding schemes, in this paper we adopt the random two-point exchange mutation operator and the random sub-fragment exchange crossover operator for the Prufer sequence. The random two-point exchange mutation method is often used in several kinds of problems. As shown in Figure 3, two random exchange points are firstly selected from a particle, and then an exchange between corresponding values in two points will be made.

**Figure 4.**
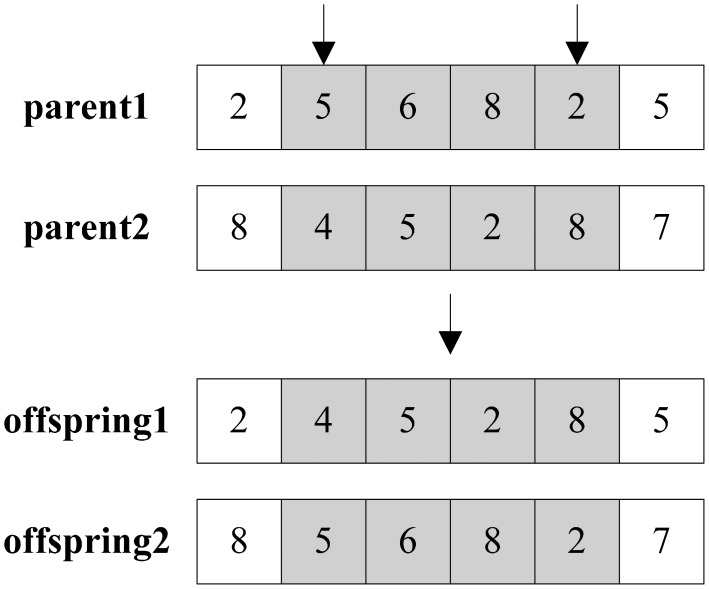
Illustration of crossover operation.

Similarly, the random sub-fragment exchange crossover firstly also selects two random points, and then exchanges corresponding values between two points from one parent particle to the other one. The operation is illustrated in Figure 4.

#### 4.2.3. Fitness Function

The mutation and crossover operators can not only preferably maintain the population diversity, but also make offspring population maintain the preferable characteristics. As in nature, the difficulty of DPSO is to provide the possible driving mechanism for better individuals to survive.

Evaluation is to associate each individual with a fitness value that reflects how good it is based upon its achievement of the objectives. The higher the fitness value of an individual is, the higher its chances of survival, reproduction and its representation in the subsequent generation are.

As the problem (9) is a single-objective optimization one, we just use the energy consumed by the constructed tree, namely Equation (8), to evaluate relative merits of each particle, but the problem (10) is a multi-objective optimization one, so we cannot merely use single objective value like energy or lifetime to evaluate the particle. To our knowledge, there are a lot of methods to deal with multi-objective optimization problems such as the weighted-sum method, the compromise approach method, the utility-function method and so on. In this paper, the phenotype sharing function with Pareto conception [37] is adopted to comprehensively compute the fitness of the particle:

*Definition 4 (Pareto Dominance)*: A vector *v* = (*v_1_, v*_2_, …, *v_n_*) is said to dominate *u* = (*u_1_, u_2_*, , *u_n_*) (denoted by *u* ≺ *ν*) if ∀*i*∈ {1,2,⋯,*n*}, *u_i_*≤ *ν_i_* ∧ ∃*i* ∈ {1,2,⋯, *n*},*u_i_* < *ν_i_*. Based on Definition 4, a feasible solution *v* is said to be non-dominated with respect to the set *Ω*, if there does not exist another *u*^∈^ *Ω* such that *u* ≻ *ν*. Furthermore, the feasible solutions that are non-dominated within the entire search space are called the Pareto optimal solutions. In this paper we call the particle *i* ≻ *j* when there satisfies that:

(17) $$\begin{array}{c} l\left(\text{tree}\left(i\right)\right)\geq l\left(\text{tree}\left(j\right)\right)\\ E\left(\text{tree}\left(i\right)\right)\leq E\left(\text{tree}\left(j\right)\right)\\ \left|E\left(tree\left(i\right)\right)-E\left(\text{tree}\left(j\right)\right)\right|+\left|l\left(\text{tree}\left(i\right)\right)-l\left(\text{tree}\left(j\right)\right)\right|\neq 0 \end{array}$$

where *tree*(*i*) represents the route tree structure decoded from the particle *i*.

*Definition 5 (Target Distance fd_ij_): fd_ij_* is the distance between the two particles *i* and *j*. Supposed that the distance has *m* dimensions which are noted as *f_1_d_ij_, f_2_d_ij_*, …, *f*_m_*d_ij_* respectively, and:

(18) $$fd_{ij}=f_{1}d_{ij}+f_{2}d_{ij}+\ldots +f_{m}d_{ij}=\left|f_{1}\left(x^{i}\right)-f_{1}\left(x^{j}\right)\right|+\left|f_{2}\left(x^{i}\right)-f_{2}\left(x^{j}\right)\right|+\ldots +\left|f_{m}\left(x^{i}\right)-f_{m}\left(x^{j}\right)\right|,i\neq j.$$

*Definition 6 (Dominance Measure D(i)): D*(*i*) denotes the state of domination the *i*-th particle with respect to the current population, and:

(19) $$D\left(i\right)=\sum_{j=1}^{p}nd\left(i,j\right)$$

where *nd*(*i,j*) equals to one if particle *j* dominates particle *i*, and zero otherwise.

*Definition 7 (Sharing Function sh(fd_ij_))*:

(20) $$sh\left(fd_{ij}\right)=\{\begin{array}{cc} 1, & if\;fd_{ij}\leq \sigma_{s}\\ 0, & \text{otherwise} \end{array}$$

where *σ_s_* is a sharing parameter.

*Definition 8 (The Neighbor Density Measure N(i)): N(i)* associated with particle i is defined as:

(21) $$N\left(i\right)=\sum_{j=1}^{p}sh\left(fd_{ij}\right)$$

*Definition 9 (Fitness Function F(i))*: The fitness of a given particle *F*(*i*) can be defined as follows:

(22) F(i)=(1+D(i))×(1+N(i))

However, problem (10) is more than a nonlinear constrained multi-objective optimization problem. The actual energy consumption of some particles after decoding may exceed the energy consumption constraint, so here we further introduce the penalty function combined with the phenotype sharing function to convert it into a non-constrained one.

*Definition 10 (Penalty Function P(i, Q)): P*(*i, Q*) = *T*(*i*) + *Q* × *S*(*i*), where *Q* × *S*(*i*) is a penalty item and *Q* is a penalty factor whose limit is ∞. The penalty function method is a widely used and effective optimization method. Its basic idea is to greatly punish those iteration points which attempt to violate constraints during the process of solving the constrained optimization problem, thus converting the constrained one to the non-constrained one. Due to the energy consumption constraint, in this paper we further combine the penalty function to evaluate the merits of particles. In order to achieve more Pareto feasible solutions and improve the convergence speed, here we propose four different penalty functions, indicated as follows, which are all constructed by the additional method and have different effect.

*Penalty Function 1 (P1)*: In this penalty function, as shown in Equation (23), the penalty factor is a 2-based exponential function, whose index is the ratio between the current solution (energy consumption of the particle) and the feasible solution (maximal permissible energy consumption). While *T*(*i*) and *S*(*i*) are both set as *F*(*i*). The more the obtained solution deviates from a feasible solution, the greater the degree of punishment will be. It grows exponentially with the intensity deviation:

(23) $$F'\left(i\right)=\left(1+2^{pf}\right)\times F\left(i\right)$$

where *T*(*i*) = *F*(*i*) = *S*(*i*), *Q* = 2*^pf^*, $pf=\left\lfloor \frac{E\left(G'\right)}{\varepsilon E\left(G^{*}\right)}\right\rfloor$.

*Penalty Function 2 (P2)*: In this penalty function, as shown in Equation (24), we directly select the difference between the current solution and the feasible solution as the penalty item when energy consumption exceeds the constraint, while *T(i)* is set as *F(i)*:

(24) $$F'\left(i\right)=F(i)+{\left[\max(pf,0)\right]}^{2}$$

where *S*(*i*) = [max(*pf*, 0)]^2^, *pf* = *E*(*G*′) − *εE*(G*) and *T*(*i*) = *F*(*i*).

*Penalty Function 3 (P3)*: In this penalty function, as shown in Equation (25), the penalty factor is a quadratic function, whose cardinal number is the ratio between the current solution and the feasible solution. *T*(*i*) and *S*(*i*) are both set as *F*(*i*). Compared to P1, the growth trend of this kind of punishment is relatively stable with the degree of deviation:

(25) $$F^{\prime }\left(i\right)=\left(1+pf^{2}\right)\times F\left(i\right)$$

where *T*(*i*) = *F*(*i*) = *S*(*i*), *Q* = 2*pf*^2^, $pf=\left\lfloor \frac{E\left(G'\right)}{\varepsilon E\left(G^{*}\right)}\right\rfloor$.

*Penalty Function 4 (P4)*: This penalty function, as shown in Equation (26), takes a logarithmic function method to construct the penalty factor. *T*(*i*) and *S*(*i*) are both set as *F*(*i*). What the difference between the P1 and P3 is the extent of punishment. The punishment to the particle grows slowly by the deviation of the current solution:

(26) $$F^{\prime }\left(i\right)=\left(1+\ln\left(1+pf\right)\right)\times F\left(i\right)$$

where *T*(*i*) = *F*(*i*), *S*(*i*) = *F*(*i*), *Q* = ln(1 + *pf*) and $pf=\left\lfloor \frac{E\left(G'\right)}{\varepsilon E\left(G^{*}\right)}\right\rfloor$.

Although the energy consumption of some particles after decoding exceeds the permitted range, they may carry some optimal information. Instead of eliminating particles that violate the constraints before the next iteration, the penalty-based fitness function retains these particles in order to give them some opportunities to have their optimal information inherited in later iterations. Thus, the penalty-based fitness function not only evaluates the merits of particles, but also has part of optimal information carried by particles preserved to allow populations to maintain a high diversity.

The value of Equations (23)–(26) may be multi-value during the search process of multi-objective PSO, and particles often possess more than one global best value and personal best value which are preserved in an external archive in general. A proper mechanism of choosing leader particles can help find more Pareto solutions in shorter time, so it is important to decide how to choose the leader particles to direct the movement of particles. In order to avoid the external archive from growing too big, we adopt ε-dominance [38] to reduce the external archive.

### 4.3. Algorithm Overview

Our proposed algorithm consists of Procedure1 and Procedure 2. In Procedure 1, we propose the DPSO algorithm where the notions of mutation and crossover operators in genetic algorithm are incorporated. The integration can not only keep the diversity of population, but also make offspring population maintain preferable characteristics.


**Procedure 1: DPSO Algorithm for Near-optimal Subgraph**
G*
**Step1:** Initialize network, swarm and relative parameters;**Step2:**
*Iteration* = 1;**Step3:** Get particle *X_it_* by operations of *Mutation, SelfCross* and *SocialCross*;**Step4:** Construct routing tree by particle *X_it_* and compute energy consumption;**Step5:** Update *pbest_i_* and *gbest_i_* if necessary;**Step6:**
*Iteration*++;**Step7:** If *iteration* < *iterationMax*, go to Step3;**Step8:** Output results *E*(G*).


In the Procedure 2, we further take into account the nodal remaining energy combined with the output result *E*(G*) from Procedure 1. Since it is a multi-objective optimization problem, we design the fitness function with the phenotype sharing function. Moreover, with the purpose of achieving more Pareto feasible solutions and improving the convergence speed, we introduce the penalty function to transform the constrained optimization problem into solving unconstrained optimization problems.


Procedure 2: DPSO Algorithm for Optimizing Network Lifetime by Applying *E*(G*)
**Step1:** Initialize network, swarm and relative parameters from the **Procedure 1** output result.**Step2:**
*Runtime* = 1;**Step3:** Select leader particles;**Step4:**
*Iteration* = 1;**Step5:** Get particle *X_it_* by operations of *Mutation, SelfCross* and *SocialCross*;**Step6:** Compute the fitness of particle *X_it_* by using the phenotype sharing function and the penalty function;**Step7:** Update leader particles;**Step8:**
*Iteration*++;**Step9:** If *iteration* < *iterationMax*, go to step5;**Step10:** Randomly select a Pareto solution to compute each node's residual energy;**Step11:**
*Runtime*++;**Step12:** If *runtime* < *runtimeMax*, go to step3;**Step13:** Output results;


*Lemma 1*: Assume that *popsize* is the number of particles, and each particle's length is *n*. We set the PSO maximum generation number *eval* as the convergent indicator. During each round of the PSO iteration, the maximum number of iterations is *R*. The complexity of the algorithm in the experiment I is *O*(*R* × (*popsize* + *eval*) × *nlogn*). In experiment II, we can conclude the complexity is *O*(*R* × (*popsize* + *eval* × *nlogn* + *R* × *eval* × *popsize*2).

*Proof*: In experiment I, the PSO algorithm conducted several rounds of iteration. The maximum number of iterations is *R*. In each round of the PSO iteration, the maximum number of iterations is *eval*. The complexity of decoding particles is *O*(*nlogn*). Therefore, by combining the results of two iterations it can be drawn the last time that the final complexity is *O*(*R* × (*popsize* + *eval*) × *nlogn*). In experiment II, the fitness function of the particles is changed. In each PSO iteration, the algorithm adds the way of choosing the optimal particle, so the time complexity is also changed this way. The time complexity of each round of the PSO iteration is *eval* × (*O*(*nlogn*) + *popsize*2 + *popsize* × *logpopsize*, so the total complexity is *OR*×*popsize*+*eval*×*nlogn*+*R*×*eval*×*popsize*^2^.

## 5. Experimental Study

In this section, the performances of the improved discrete PSO method applied to network optimization in the aspects of energy and lifetime are observed through lots of simulations, which are implemented in MATLAB. We compare the proposed algorithms with other previous tree routing algorithms, such as SPT, MST, SLT and Greed Steiner, with respect to several metrics.

Without lost of generality, we generate 50 sensor nodes randomly distributed in a 50 m × 50 m region with *k* source nodes and one sink node. We initialize each relative parameter as listed in Table 1.

### 5.1. Route Constructure in Consideration of Energy Consumption

**Figure 5.**
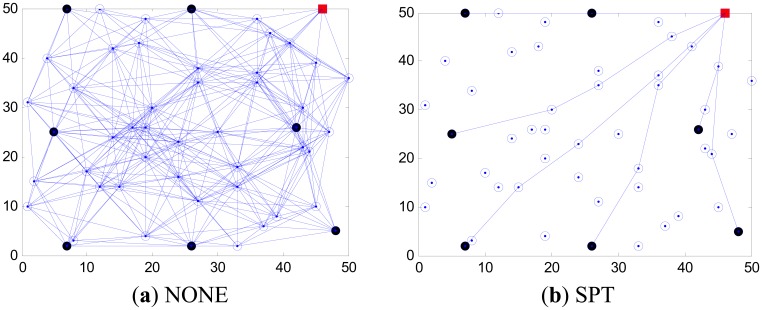

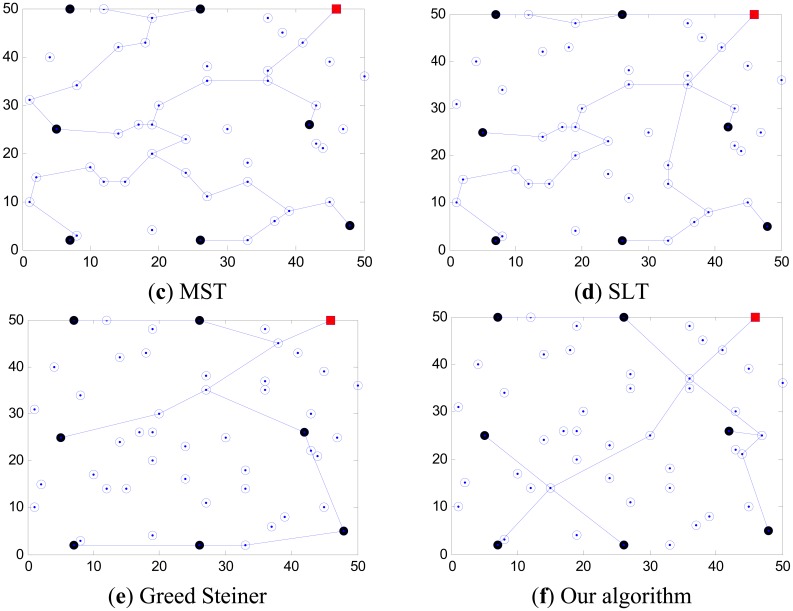
Routing tree structures.

In this simulation, we consider how to construct an optimal routing tree with respect to minimum energy consumption. Firstly, we set *k* to be 7, *r_c_* to be 20 m and *q* to be 80 nJ/bit to simulate a network, and the tree structures derived by SPT, MST, SLT, Greed Steiner and our algorithm are shown in Figure 5. In each picture, the red solid square, black solid circles and hollow circles respectively represent the sink node, source nodes and other relay nodes. Data aggregation occurs where information streams intersect.

From Figure 5, our algorithm outperforms STL, MST and SLT, which few nodes participate in the connection of network in the sense that it improves the utilization ratio of the network. What is more, the edges formed in the network are obviously less than in the results of other algorithms. All these contribute to reducing the communication energy, improving the network stability and effectively extending the network lifetime.

**Figure 6.**
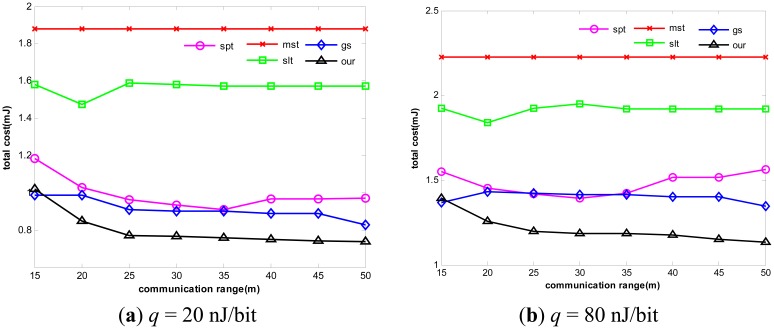
Impact of *r_c_* to energy consumption when *k* = 7.

**Figure 7.**
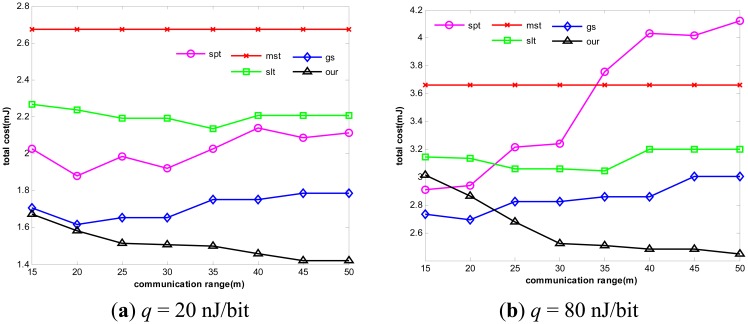
Impact of *r_c_* to energy consumption when *k* = 15.

However, the routing structure of our algorithm is slightly different from the one derived by Greed Steiner, because Greed Steiner obtains the global optimal solution through the local optimal solution. What distinguishes our algorithm is that it maintains excellent global optimization ability. The detail of the relation can be seen in Figures 6 and 7.

In Figure 6, we set *k* to be 7 and *q* to be 20 nJ/bit and 80 nJ/bit respectively. By varying *r_c_* from 15 m to 50 m, we can control the connectivity of the network. In Figure 7, we change network structure with *k* to be set to 15 and *q* set to 20 nJ/bit and 80 nJ/bit respectively. The results are as follows:

Without considering aggregation cost in the process of tree construction, the energy consumption of Greed Steiner is still relatively high. As expected, our algorithm almost outperforms all other algorithms in different communication ranges. It can adapt itself to a variety of situations. In contrast to other algorithms, our algorithm can dynamically change route selection and decide to select which nodes to perform data aggregation according to different network structures and average unit aggregation costs, and it can effectively trade off multi-hop relay benefiting from high data reduction ratio and single-hop transmission benefiting from less unit aggregation cost.

### 5.2. Route Constructure in Consideration of a Trade-off between Energy Consumption and Lifetime

In this simulation, we further consider nodal remaining energy. We fix *r_c_* to 50 m, and set *k* to be 7 and *q* to be 20 nJ/bit and 80 nJ/bit respectively.

**Figure 8.**
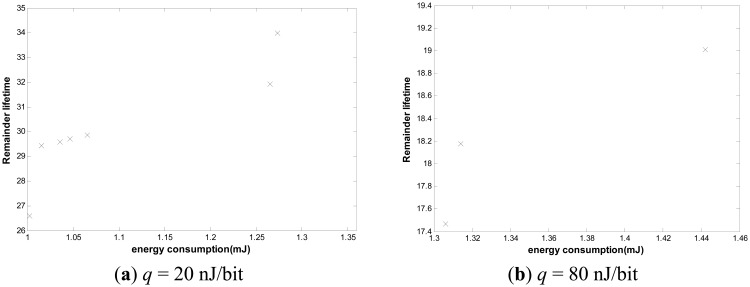
Pareto Front when ε = 1.5.

As previously mentioned, problem (10) is a multi-objective optimization problem and its Pareto optimal solutions are multiple. Figure 8 presents all Pareto optimal solutions in a certain stage where each node is left with different energy, with *ε* to be 1.5 and *q* to be 20 nJ/bit and 80 nJ/bit, respectively. Then, in order to achieve better Pareto optimal solutions, we further conduct experiments under four different penalty functions proposed in this paper. The experimental results are shown in Table 2 as follows. The table shows the number of the feasible solution we can get under the iteration in the case of 100, 300 and 500, when using different penalty functions and the value of *ε* changing from 1.1 to 1.8.

According to the experimental results shown in Table 2, we can see that in different iterations, the four different penalty functions exhibit different effects. After a relatively small number of iterations, for example, 100 times, the use of penalty function P1 can give more feasible solutions. As the number of iterations increases, P3 and P4 begin to gradually show better results. After 300 and 500 iterations, respectively, P3 and P4 achieve the most feasible solutions. The results illustrate well that in a relatively small number of iterations, in order to converge as soon as possible, we maximize the degree of punishment for the particles' constraint violations. With the increase of the number of iterations, we should also take into account the optimal information carried by these particles, so the more the number of iteration increases, the smaller the intensity of the punishment is.

**Figure 9.**
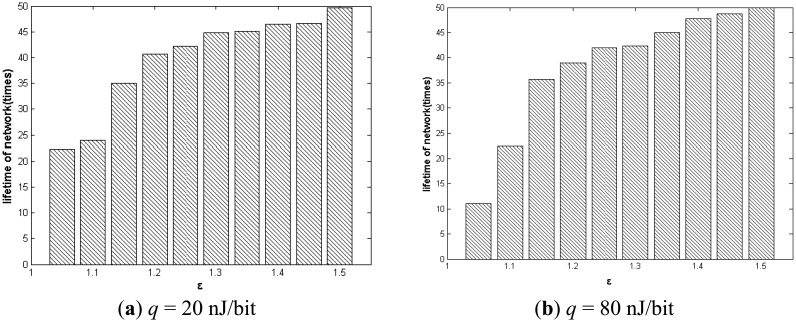
Impact of ε on network lifetime.

We can also see that when the value of *ε* is 1.5, the number of the feasible solutions is the greatest. This occurs because as the *ε* value is too big, the energy consumption constraint relaxes, and the lifetime becomes smaller. The Pareto solution requires minimal energy consumption and maximized lifetime. Furthermore, we study the impact of *ε* on the performance of our algorithm. By varying *ε* from 1 to 1.5, we can observe the change of network lifetime obviously. As shown in Figure 9, with the increase of *ε*, the network lifetime becomes longer.

**Figure 10.**
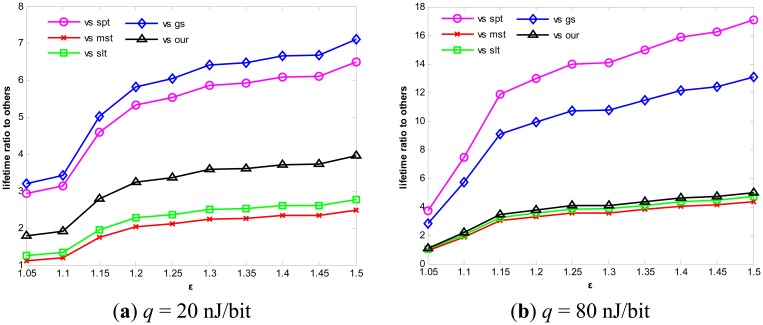
Lifetime ratio of our algorithm to other algorithms.

As we see, a small increase of ε may lead to a huge lifetime extension. We further compare our algorithm with others. As shown in Figure 10, with the increase of *ε*, the lifetime ratio of our algorithm to other algorithms increases drastically. These algorithms include not only SPT, MST, SLT and Greed Steiner, but also our algorithm adopted in the first simulation. While other algorithms use consistent nodes throughout to transmit or aggregate data, despite the fact that some nodes possess little energy and other nodes have vast energy remaining, resulting in some nodes' premature death, our algorithm can adjust itself to select an optimal route which can balance the total energy consumption and nodal remaining energy, effectively extending the network lifetime.

## 6. Conclusions

In this paper, we mainly describe the impact of the energy and lifetime in WSNs on the establishment of a reliable network. We design discrete PSO-based algorithms to construct an optimal routing tree structure in the process of data gathering with aggregation, considering not only communication cost, but also aggregation cost. In addition, we further adaptively adjust route strategy according to the remaining energy in each node. This is not only a multi-objective optimization problem, but also a non-linear constrained optimization problem. In this paper, combined with the phenotype sharing function and penalty function, we design heuristic algorithms based on DPSO to solve the above problems. The simulation results show that our algorithms can provide a route structure with lower energy consumption while considering aggregation cost, and can trade off communication load and lifetime while further considering nodal remaining energy. It ensures that the network will work properly and securely. In future work, we will pursue optimization of other additional aspects of performance regarding other security requirements while constructing route structures, such as delay and fault-tolerant ability.

## Figures and Tables

**Table 1.** Parameter table.

**Symbol**	**Definition**	**Value**
*σ_s_*	A sharing parameter whose dimensions equal to the number of objectives	[0.01 0.01]
*α*	The energy consumed by sending each bit of data	50 nJ/bit
*β*	The energy consumption in the amplification circuit for forwarding each bit of data	100 pJ/bit/m^2^
*w_0_*	The data amount sent by each source node	400 bit
*r_s_*	The correlation range	50 m
*ρ*	The correlation coefficient between two nodes in an approximated spatial model	*ρ* = 1 − *d/r_s_* while *d* < *r_s_, ρ* = 0 otherwise
*r_c_*	The maximum communication range of each sensor node	From 15 m to 50 m
*n*	Number of nodes	50
*k*	Number of source nodes	7 and 15
*q*	Average unit aggregation cost	20 nJ/bit and 80 nJ/bit
*ε(epsilon)*	Maximal permissible times of energy consumption of *G*′ to G*	From 1 to 1.5
*E_r*	The initial energy of each relaying node	2 mJ

**Table 2.** The number of Pareto optimal solutions obtained by four different penalty functions.

	***ε* = 1.1**			***ε* = 1.2**			***ε* = 1.3**			***ε* = 1.4**		
			
	**100**	**300**	**500**	**100**	**300**	**500**	**100**	**300**	**500**	**100**	**300**	**500**
**P1**	5.61	5.65	5.87	5.82	5.82	5.95	5.38	5.61	5.88	5.61	5.78	6.10
**P2**	4.17	4.88	4.89	4.32	4.56	5.00	4.16	4.61	5.03	4.41	4.51	5.23
**P3**	5.23	5.73	5.82	5.54	5.86	5.88	5.43	5.71	5.95	5.36	6.02	6.11
**P4**	5.16	5.49	6.65	5.64	5.80	5.96	5.31	6.21	6.22	5.58	5.75	6.26
	***ε* = 1.5**			***ε* = 1.6**			***ε* = 1.7**			***ε* = 1.8**		
			
	**100**	**300**	**500**	**100**	**300**	**500**	**100**	**300**	**500**	**100**	**300**	**500**

**P1**	5.83	5.90	6.11	5.65	5.80	6.01	5.71	5.85	5.96	5.66	5.72	5.95
**P2**	4.43	4.66	5.40	4.48	4.74	4.86	4.33	4.55	4.80	4.37	4.68	4.69
**P3**	5.73	6.09	6.13	5.64	5.85	6.03	5.77	5.86	5.90	5.64	5.81	5.86
**P4**	5.50	5.63	6.35	5.62	5.68	5.78	5.54	5.80	5.99	5.63	5.77	5.97
